# Impacts of grandparenting on older Chinese adults’ mental health: a cross-sectional study

**DOI:** 10.1186/s12877-023-04396-x

**Published:** 2023-10-13

**Authors:** Huan Wang, Jianyuan Huang

**Affiliations:** Institute of Population Research, Hohai University, No.8 Focheng Road, Nanjing, Jiangsu Province China

**Keywords:** Depressive symptoms, Grandparenting, Intergenerational support, Cross-sectional data, China

## Abstract

**Background:**

The practice of grandparenting has been growing in popularity worldwide, particularly in Asian societies. Nevertheless, there is a lack of thorough studies investigating the mental health effects of grandchild care on grandparents, particularly within the family context. The present study aimed to explore the impact of grandparenting on depressive symptoms in older Chinese adults, taking into account the functional role of intergenerational support.

**Methods:**

Using the China Longitudinal Aging Social Survey (CLASS, 2014 and 2018, N = 9,486), we employed the Pooled Ordinary Least Square method (POLS) to explore the association between depressive symptoms and grandparenting intensity as well as include the interaction terms to investigate the role of intergenerational support among grandparents aged from 60 to 80.

**Results:**

After adjusting for control variables, both non-intensive (-0.17; 95% CI: -0.30, -0.03) and intensive (-0.69; 95% CI: -0.95, -0.43) childcare, as well as giving financial support to adult children (-0.06; 95% CI: -0.08, -0.04) and emotional closeness with them (-0.94; 95% CI: -1.15, -0.72), were found to have a positive impact on the mental health of grandparents. Giving financial support (non-intensive: -0.04, 95% CI: -0.07, -0.01; intensive: -0.06, 95% CI: -0.13, -0.01) and providing instrumental support to adult children (non-intensive: -0.12, 95% CI: -0.24, -0.01; intensive: -0.19, 95% CI: -0.37, -0.02) moderated the association between grandparenting and the mental health of older adults, regardless of care intensity. However, the moderating role of receiving financial support (-0.07; 95% CI: -0.12, -0.02) only existed when non-intensive childcare was provided.

**Conclusion:**

Grandchild care predicted better mental health in grandparents, mainly when they engaged in intensive grandparenting. Emotional closeness and providing financial support to adult children brought mental health benefits to grandparents involved in childcare. Giving financial support and providing instrumental support to adult children moderated the association between grandparenting and the mental health of older adults. However, the moderating role of receiving financial support from adult children only existed when non-intensive childcare was provided.

**Supplementary Information:**

The online version contains supplementary material available at 10.1186/s12877-023-04396-x.

## Introduction

As older adults across the globe live longer and healthier lives, they have more opportunities to become grandparents providing care for their grandchildren [1]. Simultaneously, enhancing older adults’ mental health and psychological well-being has become an imperative policy objective due to the accelerating global phenomenon of population aging. However, it is still unclear whether and how grandparenting is associated with grandparents’ health [2].

Existing research presents varying views on grandparenting’s health effects, noting positive, negative, and neutral impacts [3–7]. Discrepancies may stem from diverse data, measurement methods, methodologies, and cultural contexts [8–11]. Role theories often explain the health effects of grandparenting. Role enhancement theory suggests grandparenting can boost mental health by compensating for lost social roles and increasing self-efficacy and life satisfaction [12, 13]. Role strain theory, however, argues that caregiving may be burdensome for older adults due to their existing responsibilities [14, 15]. Health impacts may be a mix of these theories, depending on the grandparent involvement level [16, 17]. Increased caregiving intensity was found to amplify depression symptoms in the U.S. and European grandparents while improving psychological well-being in Asian grandparents [18–20], mainly due to cultural differences. These inconsistent results underscore the importance of considering care intensity when examining this relationship, an aspect often overlooked in current research.

Grandchild care involves meaningful interaction and mutual support between generations, which is fundamental in fostering intergenerational solidarity [21]. Existing studies have noted the intricate dynamics of intergenerational support, encompassing financial, instrumental, and emotional dimensions, and their implications for the mental health of older adults [22, 23]. Financially, while receiving support can enhance life satisfaction and mitigate depressive symptoms in the elderly, providing financial aid to adult children might either fortify their self-efficacy or augment their economic strain and stress [24–26]. Instrumentally, older adults can derive a sense of purpose from tasks like assisting with grandchild care or household chores, yet excessive involvement might lead to frustration or foster feelings of uselessness due to the time-intensive nature of such support [27]. Emotionally, a predominant consensus highlights the benefits of receiving emotional support for older adults, marked by reduced depression and improved mental health [28]. However, this can be counteracted when the support is either scarce or excessive. On the provision side, while offering emotional support can manifest the wisdom and values of the elderly, bolstering self-affirmation, an overemphasis on attending to adult children’s emotional needs might be perceived as a reflection of their parenting failure, intensifying stress among the senior population [23, 29]. Hence, the complex interaction between intergenerational support and health calls for further investigation, particularly within the context of grandparenting.

According to the stress process model, the impact of childcare-related health outcomes for grandparents is influenced by various factors, such as social support, socioeconomic status, and cultural norms [30–32]. Following this point, the associations between grandparenting and mental health may vary in terms of strength and direction depending on the level of intergenerational support. For instance, a recent investigation focusing on Mexican American grandparents revealed that when moderate or low financial support was extended to adult children, engaging in grandparenting activities was associated with a lower incidence of depressive symptoms [33]. Conversely, when higher levels of financial support were provided to adult children, grandparenting was linked to a higher occurrence of depressive symptoms. Notably, this study did not find any significant interaction effects between receiving financial support from adult children or emotional support and grandparenting on depressive symptoms. In Taiwan, older caregivers receiving satisfactory emotional support from adult children had fewer depressive symptoms than the non-caregiver’s cohort, and they also found no significant health impacts of financial and instrumental support [34]. Some studies also indicated that not all intergenerational support brought health benefits for grandparents, specifically when that support was less desirable and expected [35]. The studies mentioned above suggested that the type, direction, and intensity of intergenerational support may impact the connection between grandparenting and the health of older adults.

Given China’s rich cultural heritage and rapid demographic transition, the country emerges as a compelling case study for exploring the nexus between grandparenting, intergenerational support, and older adults’ mental health. As of 2020, 18.70% of China’s population was aged 60 and above, projected to rise to 34.6% by 2050 [36]. Concurrently, the fertility rate has plummeted from 5.8 to 1970 to 1.3 in 2020 [37], resulting in a burgeoning elderly population and a shrinking younger generation. However, this evolving demographic landscape presents a paradox: an expanding pool of grandparents coincides with fewer opportunities for grandparenting due to the declining younger generation.

Traditional Confucian values have shaped Chinese families, emphasizing adult children’s responsibility to support their parents in later life and the active involvement of older adults in caring for their grandchildren [38]. However, contemporary Chinese families are witnessing a transformation in intergenerational relations. Socioeconomic advancements have catalyzed a shift from the traditional one-way support model from adult children to older parents towards a reciprocal model emphasizing mutual assistance across generations [16, 17, 38]. This shift profoundly impacts family decision-making, resource allocation, and the significance attributed to familial roles and actions. Regrettably, existing research in the Chinese context has seldom integrated these intergenerational shifts when examining the health implications of grandchild care [26]. Therefore, a comprehensive exploration synthesizing cultural, demographic, and intergenerational perspectives is paramount to unraveling the complex relationship between grandparenting and mental health in China.

This study aimed to investigate the correlation between grandparenting and mental health and explore the role of intergenerational support in shaping this relationship. Based on previous research and taking into account traditional Chinese culture, we propose the following hypotheses.

H1. Engaging in grandparenting is linked to improved mental health among older Chinese adults.

H2. Grandparents experience better mental health when they receive support from their adult children rather than when they provide support.

H3. Intergenerational support moderates the relationship between grandparenting and mental health outcomes.

## Methods

### Data

The analysis used data from the CLASS, a nationally representative and large-scale survey. The objective of the CLASS is to collect data on the living and health status of Chinese adults aged 60 years and above. The CLASS was initially conducted in 2014 and continued in 2016 and 2018. We utilized the first and third waves of the CLASS survey (2014 and 2018) due to the lack of grandparenting data in the 2016 wave. The sample in 2014 consisted of 11,511 cases, and 11,418 observations in 2018, with 4,346 cases being re-observed. In this sample, we excluded 9,054 observations of older adults who did not have any grandchildren in either 2014 or 2018. Following previous studies [39, 40], we excluded adults over the age of 80 (1,058 observations) since only 4.73% of individuals in this age group provided childcare. We excluded respondents who had at least one functional ability impairment (= 3,331 observations) because severe health decline may limit their ability to provide care and lead to more depressive symptoms [4, 14]. Finally, our analysis included 9,486 person-year observations, with 4,513 observations in 2014 and 4,973 observations in 2018. Only 32.53% of the sample we studied in 2014 were present in 2018, resulting in an imbalanced panel dataset for our model regression. The Research Ethics Committees of Renmin University of China granted ethical approval for all CLASS waves. All participants gave informed consent.

### Measurements

#### Depressive symptoms

Depression is an important indicator of mental health and occurs more often in later life [41]. The number of depressive symptoms was measured using a 9-item Chinese version adapted from the 20-item Center of Epidemiological Studies Depression (CES-D) Scale [42]. This 9-item scale inquiries about participants’ feelings and the physical sensations experienced in the past week. It includes questions about feeling happy, lonely, and upset, enjoying life, having a poor appetite, sleeping troubles, feeling useless, having nothing to do, and enjoying life. It has been validated in several studies [3, 43]. Respondents were required to rate the frequency of depressive symptoms they have experienced in the past week on a three-point scale (none = 0, some of the time = 1, most of the time = 2). After reverse-coding the positive items, we create a cumulative score ranging from 0 to 18 with Cronbach’s alpha of 0.78 in our 2014 sample. A higher score reflects greater depressive symptoms and a poorer mental state.

#### Grandparenting

This study measured grandparenting based on the intensity of care as a time-variant variable. Grandparents were asked if they provided childcare for their grandchildren. If the answer was “yes,“ they were then asked to indicate the number of hours they spent per day on average caring for each grandchild over the past 12 months. The response options were on a 6-point scale, ranging from “none” (0) to “more than eight hours” (5). Since grandparents may have more than one grandchild, we categorized grandparenting into three types based on the average time spent caring for all grandchildren: non-caregivers (the reference group who do not provide any care), non-intensive caregivers (those whose average care points are ≤ 4), and intensive caregivers (those whose average care points are > 4).

#### Intergenerational support

Three time-varying variables represented intergenerational support between grandparents and adult children, including mutual financial, instrumental, and emotional support.

***Financial support***. Grandparents were asked whether they received financial or in-kind support, such as food or gifts, from each of their adult children during the past year. They were asked to choose from the nine equivalent amount categories in Chinese RMB currency. Similarly, grandparents were asked whether they provided financial or in-kind support to their adult children based on the same set of questions. This study used the averaged median value across adult children’s responses. This value was then logged (+ 1) to improve the skewed distribution of the variables in our final analysis.

***Instrumental support.*** Receiving/giving instrumental support was measured by asking grandparents whether they received/gave any help from/to their adult children over the previous year. Response codes ranged from 0 (= almost none) to 4 (= almost daily). Our analysis used averaged score points to measure the instrumental support to/from grandparents across adult children. A higher score implies a higher instrumental support level.

***Emotional support.*** Emotional support was measured by a single item asking grandparents to rate the quality of their relationship with their adult children on a 3-point scale (0 = not close, 1 = moderate, 2 = close). A higher score indicates better emotional relations. We used averaged scores across adult children in the final analysis.

***Other control variables***. We controlled for demographic and socioeconomic factors, as well as health conditions, in our regression models. This included considering covariates that were linked to depressive symptoms. These factors included gender (0 = female), age group (0 = 60–69 years old), marital status (0 = unmarried), living arrangements (0 = live without adult children), residential region (0 = rural region), education (0 = no education or less than elementary school), log-transformed household income (log(+ 1)), working status (0 = no), social activity (0 = no), pensions (0 = no), chronic diseases (0 = no), and self-rated health (0 = unhealthy). Additionally, we included a dummy variable for the wave year.

### Analysis strategy

Descriptive analysis was processed to examine the demographic, socioeconomic, and health background of the grandparents. Percentages represented categorical variables, while continuous variables were described by mean (standard deviation, S.D.). Analysis of variance (ANOVA) and Chi-square test was performed to explore the differences between non-caregivers, non-intensive caregivers, and intensive caregivers. We used multiple imputations with chained equations to create 20 data sets and correct missingness.

Using Pooled Ordinary Least Squares (POLS) regression models, we initially examined the association between grandparenting and mental health outcomes, while adjusting for control variables (base model). Each type of intergenerational support variable was then added sequentially to this base model. After integrating all support variables, we formed the full model. Moderating effects were evaluated by adding interaction terms between grandparenting and intergenerational support to the base model based on different types of support. Finally, all interaction terms were incorporated into the base model. The year dummy variable was incorporated to account for unobserved time-related characteristics.

The POLS model has the advantage of capturing the between-individual variations, and including a year dummy variable can help control for unobserved time-specific variations. However, one limitation of POLS is that it may not fully account for the unobserved time-constant characteristics associated with grandchild care provision [44]. An alternative approach to address this issue for longitudinal data is to use a fixed effects (FE) model. The FE model can identify within-individual changes over time and eliminate unobserved stable individual heterogeneity [34, 39]. However, the FE model may not accurately estimate when respondents have limited variation in grandparenting or are only observed once during the study period [4]. This situation applies to about 90.78% of our sample, leading to huge information loss. Hence, we mainly presented the POLS estimation results in this study and the findings of the FE model were discussed below.

## Results

### Descriptive results

Table 1 presented the descriptive statistics for all variables analyzed in the pooled sample of grandparents, categorized by caregiving intensity. Over half of the grandparents (55.60%) identified themselves as caregivers. Among grandparents, 48.39% provided non-intensive childcare, while only 7.21% provided intensive childcare. Grandparents who did not provide childcare reported the highest score for depressive symptoms (6.37), followed by non-intensive caregivers (5.80). Grandparents who provided intensive caregiving had the lowest scores for depressive symptoms (3.98). Non-intensive childcare providers had the closest emotional relationships (1.89) with their adult children compared to other grandparents, although there were slight differences between groups. Intensive childcare providers engaged in an extensive reciprocity of instrumental support resources with their children. Intensive childcare givers offered the most economic support to their adult children, while non-intensive childcare givers received the most financial support from their adult children. Robust intergenerational bonds existed, characterized by adult children providing greater support to the elderly than vice versa. Younger, married, educated, and rural grandparents were more likely to provide childcare. Grandchild caregivers reported better self-rated health, fewer chronic diseases, and a more satisfying life.

**Table 1:** Descriptive statistics of the pooled grandparent samples 2014–2018 (M(SD)/%)

	ALL	Non- caregivers	Non-intensive caregivers	Intensive caregivers	Chi-square/ F-Values
**Depressive symptoms** (0–18)	5.92 (3.85)	6.37 (3.42)	5.80 (3.49)	3.98 (3.18)	147.92^***^
Sex (ref = female)	54.32	54.99	53.83	53.51	1.37
Age group (ref = 60–69)	26.98	33.40	22.77	15.64	174.24^***^
Marital status (ref = non- married)	79.57	77.94	80.33	84.50	18.71^***^
Living arrangement (ref = live without adult children)	44.53	25.59	55.82	85.38	130.13^***^
Residential region ( ref = rural region)	63.92	59.57	65.90	77.34	95.83^***^
Social activity (ref = no)	33.40	33.12	33.70	33.04	0.38
Pension ( ref = no)	79.11	77.78	80.00	81.29	8.68^**^
Education (ref = no education or less than elementary school)	83.07	81.39	84.05	86.84	18.56^***^
Working ( ref = no)	28.00	29.58	27.47	21.78	18.97^***^
Emotional closeness (0–2)	1.88 (0.32)	1.87 (0.32)	1.89 (0.31)	1.88 (0.34)	3.05^**^
Giving money to adult children (log (+ 1))	2.92 (3.42)	2.62 (3.31)	3.10 (3.43)	3.60 (3.77)	36.28^***^
Receiving money from adult children (log(+ 1))	6.31(2.52)	6.19 (2.52)	6.43 (2.42)	6.19 (3.11)	11.10^***^
Giving instrumental support to adult children	0.98 (1.29)	0.56 (0.99)	1.20 (1.32)	2.05 (1.69)	84.59^***^
Receiving instrumental support from adult children	1.55 (1.26)	1.37 (1.18)	1.69 (1.27)	1.71 (1.52)	76.38^***^
Chronic conditions (ref = no)	0.68 (0.47)	0.69 (0.46)	0.67 (0.47)	0.64 (0.48)	10.61^***^
Self-rated health (ref = unhealthy)	0.86 (0.35)	0.84 (0.37)	0.87 (0.33)	0.85 (0.36)	11.51^***^
Life satisfaction (1–5)	2.03 (0.86)	2.09 (0.87)	2.00 (0.84)	1.91 (0.88)	20.99^***^
Observations	9486	4212	4590	684	
%	100	44.40	48.39	7.21	

**Note: M = mean; SD = Standard deviation;** %=percentage; ^*^ **p < 0.1**, ^**^ **p < 0.05**, ^***^ **p < 0.01**

### Results from POLS regressions

Table 2 presented the estimated regression coefficients of depressive symptoms predicted by grandparenting intensity from POLS models. Compared to non-caregivers, both non-intensive (-0.2; 95% CI: -0.33, -0.06) and intensive grandparenting (-0.70; 95% CI: -0.95, -0.44) were linked to lower levels of depressive symptoms (model 1), providing support for Hypothesis 1.

**Table 2:** POLS regression on the association between grandparenting and depressive symptoms, 2014–2018 (Coef. /[CI], N = 9486)

Variables	Model 1	Model 2	Model 3	Model 4	Model 5
**Grandparenting** (ref = no caregiving)					
Non-intensive	-0.20^***^	-0.19^***^	-0.16^**^	-0.19^***^	-0.17^**^
	[-0.33, -0.06]	[-0.32, -0.06]	[-0.29, -0.03]	[-0.33, -0.06]	[-0.30, -0.03]
Intensive	-0.70^***^	-0.71^***^	-0.64^***^	-0.71^***^	-0.69^***^
	[-0.95, -0.44]	[-0.96, -0.46]	[-0.90, -0.39]	[-0.97, -0.45]	[-0.95, -0.43]
**Controls**					
Sex (ref = female)	-0.08	-0.09	-0.09	-0.08	-0.10
	[-0.21, 0.05]	[-0.22, 0.03]	[-0.22, 0.04]	[-0.21, 0.05]	[-0.23, 0.03]
Age group (ref = 60–69)	-0.05	-0.06	-0.06	-0.05	-0.07
	[-0.19, 0.10]	[-0.20, 0.09]	[-0.21, 0.08]	[-0.19, 0.10]	[-0.21, 0.08]
Marital status (ref = non-married)	-0.86^***^	-0.82^***^	-0.83^***^	-0.87^***^	-0.80^***^
	[-1.03, -0.69]	[-0.99, -0.65]	[-1.00, -0.66]	[-1.04, -0.70]	[-0.97, -0.63]
Living arrangement (ref = live without adult children)	-0.05	-0.04	-0.09	-0.04	-0.09
	[-0.18, 0.08]	[-0.17, 0.09]	[-0.22, 0.04]	[-0.18, 0.10]	[-0.23, 0.05]
Residential region (ref = rural region)	-0.42^***^	-0.43^***^	-0.35^***^	-0.41^***^	-0.35^***^
	[-0.57, -0.28]	[-0.57, -0.28]	[-0.50, -0.21]	[-0.56, -0.26]	[-0.50, -0.21]
Social activity (ref = no)	-0.21^***^	-0.19^***^	-0.18^***^	-0.20^***^	-0.16^**^
	[-0.33, -0.08]	[-0.31, -0.06]	[-0.30, -0.05]	[-0.32, -0.07]	[-0.28, -0.03]
Pension (ref = no)	-0.25^***^	-0.22^***^	-0.21^***^	-0.25^***^	-0.19^**^
	[-0.41, -0.09]	[-0.38, -0.07]	[-0.36, -0.05]	[-0.41, -0.09]	[-0.34, -0.03]
Education (ref = no education or less than elementary school)	-0.47^***^	-0.44^***^	-0.43^***^	-0.47^***^	-0.40^***^
	[-0.66, -0.29]	[-0.63, -0.26]	[-0.61, -0.24]	[-0.65, -0.28]	[-0.58, -0.21]
Working (ref = no)	-0.18^**^	-0.19^***^	-0.18^**^	-0.19^**^	-0.19^**^
	[-0.33, -0.03]	[-0.34, -0.05]	[-0.33, -0.03]	[-0.34, -0.04]	[-0.34, -0.04]
Chronic conditions (ref = no)	0.62^***^	0.64^***^	0.63^***^	0.62^***^	0.64^***^
	[0.50, 0.74]	[0.51, 0.76]	[0.50, 0.75]	[0.50, 0.74]	[0.52, 0.76]
Self-rated health (ref = unhealthy)	-1.54^***^	-1.48^***^	-1.50^***^	-1.54^***^	-1.45^***^
	[-1.74, -1.33]	[-1.68, -1.27]	[-1.71, -1.29]	[-1.74, -1.33]	[-1.65, -1.24]
Wave (ref = 2014)	3.61^***^	3.59^***^	3.60^***^	3.64^***^	3.59^***^
	[3.49, 3.73]	[3.47, 3.71]	[3.48, 3.72]	[3.51,3.76]	[3.47,3.72]
**Intergenerational support**					
Emotional closeness		-0.98^***^			-0.94^***^
		[-1.20, -0.77]			[-1.15, -0.72]
Giving financial support to adult children (log (+ 1))			-0.06^***^		-0.06^***^
			[-0.08, -0.04]		[-0.08, -0.04]
Receiving financial support from adult children (log (+ 1))			-0.03^**^		-0.02
			[-0.06, -0.01]		[-0.04, 0.01]
Giving instrumental support to adult children				0.03	0.04
				[-0.02, 0.09]	[-0.01, 0.10]
Receiving instrumental support from adult children				-0.07^***^	-0.04
				[-0.13, -0.02]	[-0.10,0.01]
**Constant**	6.96^***^	8.68^***^	7.16^***^	7.02^***^	8.74^***^
	[6.62, 7.31]	[8.17, 9.19]	[6.79, 7.52]	[6.67, 7.37]	[8.22, 9.26]
F	329.20***	317.60***	295.34***	288.09***	256.17***
*N*	9486	9486	9486	9486	9486

**Note** Results were combined using 20 imputed data sets. Coef. = coefficient estimation; CI = confidence interval; 95% confidence intervals in brackets; ^*^ p < 0.1, ^**^ p < 0.05, ^***^ p < 0.01

Models 2–4 included intergenerational emotional closeness and financial and instrumental support as additional factors in addition to model 1. Emotional closeness, bi-directional financial support, and receiving instrumental support to adult children were found to have a significant negative relationship with depressive symptoms. After considering all types of intergenerational support (model 5), it was found that only providing financial support (-0.06; 95% CI: -0.08, -0.04) and emotional closeness (-0.94; 95% CI: -1.15, -0.72) was associated with a decrease in depression symptoms among grandparents. Meanwhile, estimates of grandparenting remained relatively stable for both non-intensive caregivers (-0.17; 95% CI: -0.30, -0.03) and intensive caregivers (-0.69; 95% CI: -0.95, -0.43). As a result, Hypothesis 2 was partially supported.

Table 3 showed the estimates for the interaction terms between grandparenting and intergenerational support. Enhanced provision of financial (non-intensive, -0.04, 95% CI: -0.07, -0.01; intensive, -0.06, 95% CI: -0.13, -0.01) and instrumental support (non-intensive, -0.12, 95% CI: -0.24, -0.01; intensive, -0.19, 95% CI: -0.37, -0.02) to adult children was associated with lower depressive symptoms among caregivers compared to non-caregivers. Increased financial support from adult children was found to be associated with a decrease in depressive symptoms, but only among non-intensive childcare providers (-0.07; 95% CI: -0.12, -0.02). We found no significant interactions between grandparenting and either emotional closeness or instrumental support received from adult children. Our findings partially support Hypothesis 3.

**Table 3:** POLS regression on the interaction effect of intergenerational support on the association between grandparenting and depressive symptoms, 2014–2018 (Coef. /[CI], N = 9486)

Variables	Model 6	Model 7	Model 8	Model 9
**Grandparenting** (ref = no caregiving)				
Non-intensive	-0.27	0.36^**^	-0.12	0.16
	[-1.11, 0.57]	[0.00, 0.72]	[-0.34, 0.09]	[-0.73, 1.05]
Intensive	0.06	-0.43	-0.56^***^	0.46
	[-1.59, 1.70]	[-1.03, 0.17]	[-0.97, -0.14]	[-1.23, 2.15]
**Controls**				
Sex (ref = female)	-0.10	-0.10	-0.10	-0.10
	[-0.23, 0.03]	[-0.23, 0.03]	[-0.23, 0.03]	[-0.23, 0.03]
Age group (ref = 60–69)	-0.07	-0.07	-0.07	-0.07
	[-0.21, 0.08]	[-0.21, 0.08]	[-0.21, 0.08]	[-0.21, 0.08]
Marital status (ref = non-married)	-0.80^***^	-0.80^***^	-0.80^***^	-0.80^***^
	[-0.96, -0.63]	[-0.97, -0.63]	[-0.97, -0.63]	[-0.97, -0.63]
Living arrangement (ref = live without adult children)	-0.09	-0.10	-0.10	-0.10
	[-0.23, 0.05]	[-0.23, 0.04]	[-0.24, 0.04]	[-0.24, 0.03]
Residential region (ref = rural region)	-0.35^***^	-0.36^***^	-0.35^***^	-0.36^***^
	[-0.50, -0.21]	[-0.51, -0.21]	[-0.49, -0.20]	[-0.50, − 0.21]
Social activity (ref = no)	-0.16^**^	-0.16^**^	-0.16^**^	-0.16^***^
	[-0.28, -0.03]	[-0.29, -0.04]	[-0.28, -0.03]	[-0.29, -0.04]
Pension (ref = no)	-0.19^**^	-0.18^**^	-0.18^**^	-0.18^**^
	[-0.34, -0.03]	[-0.34, -0.03]	[-0.34, -0.03]	[-0.33, -0.02]
Education (ref = no education or less than elementary school)	-0.40^***^	-0.40^***^	-0.39^***^	-0.40^***^
	[-0.58, -0.21]	[-0.58, -0.21]	[-0.58, -0.21]	[-0.58, -0.21]
Working ( ref = no)	-0.19^**^	-0.19^***^	-0.19^**^	-0.19^***^
	[-0.34, -0.04]	[-0.34, -0.05]	[-0.34, -0.04]	[-0.34, -0.05]
Chronic conditions (ref = no)	0.64^***^	0.64^***^	0.64^***^	0.64^***^
	[0.52, 0.76]	[0.52, 0.77]	[0.52, 0.76]	[0.52, 0.77]
Self-rated health (ref = unhealthy)	-1.45^***^	-1.45^***^	-1.45^***^	-1.45^***^
	[-1.65, -1.24]	[-1.65, -1.24]	[-1.65, -1.24]	[-1.65, -1.24]
Wave (ref = 2014)	3.59^***^	3.59^***^	3.58^***^	3.58^***^
	[3.47,3.72]	[3.46,3.71]	[3.46,3.71]	[3.45,3.71]
**Intergenerational support**				
Emotional closeness	-0.93^***^	-0.95^***^	-0.94^***^	-0.97^***^
	[-1.25, -0.61]	[-1.16, -0.73]	[-1.16, -0.73]	[-1.29, -0.65]
Giving financial support to adult children (log (+ 1) )	-0.06^***^	-0.03^**^	-0.06^***^	-0.04^**^
	[-0.08, -0.04]	[-0.06, -0.01]	[-0.08, -0.04]	[-0.06, -0.01]
Receiving financial support from adult children (log (+ 1) )	-0.02	0.01	-0.02	0.02
	[-0.04,0.01]	[-0.02,0.05]	[-0.04,0.01]	[-0.02,0.05]
Giving instrumental support to adult children	0.04	0.05^*^	0.15^***^	0.14^***^
	[-0.01,0.10]	[-0.01,0.10]	[0.05, 0.25]	[0.05,0.24]
Receiving instrumental support from adult children	-0.04	-0.04	-0.07^*^	-0.08^**^
	[-0.09,0.01]	[-0.10,0.01]	[-0.16,0.01]	[-0.17, -0.00]
**Interactions**				
Non-intensive × Emotional closeness	0.06			0.12
	[-0.38,0.49]			[-0.32,0.56]
Intensive × Emotional closeness	-0.40			-0.47
	[-1.25,0.46]			[-1.36,0.41]
Non-intensive × Giving financial support to adult children (log (+ 1))		-0.04^**^		-0.04^**^
		[-0.08, -0.01]		[-0.07,-0.01]
Intensive × Giving financial support to adult children (log (+ 1))		-0.07^**^		-0.06^*^
		[-0.13, -0.01]		[-0.13, -0.01]
Non-intensive × Receiving financial support from adult children (log (+ 1))		-0.07^**^		-0.07^***^
		[-0.12, -0.01]		[-0.12, -0.02]
Intensive × Receiving financial support from adult children (log (+ 1))		-0.01		-0.01
		[-0.09,0.08]		[-0.08, 0.08]
Non-intensive × Giving instrumental support to adult children			-0.14^**^	-0.12^**^
			[-0.25, -0.02]	[-0.24, -0.01]
Intensive × Giving instrumental support to adult children			-0.21^**^	-0.19^**^
			[-0.39, -0.04]	[-0.37, -0.02]
Non-intensive × Receiving instrumental support to adult children			0.04	0.05
			[-0.07,0.15]	[-0.05,0.16]
Intensive × Receiving instrumental support to adult children			0.09	0.13
			[-0.09,0.28]	[-0.06,0.32]
**Constant**	8.73^***^	8.51^***^	8.73^***^	8.55^***^
	[8.04, 9.42]	[7.96, 9.06]	[8.20, 9.26]	[7.84, 9.25]
F	231.31***	231.90***	211.88***.	169.74***
*N*	9486	9486	9486	9486

**Note** Results were combined using 20 imputed data sets. Coef. = coefficient estimation; Ci = confidence interval; 95% confidence intervals in brackets; ^*^ p < 0.1, ^**^ p < 0.05, ^***^ p < 0.01

The FE model yielded similar findings to the POLS estimations (see Supplemental Tables 1–2)—however, the significance of grandparenting diminished. Providing financial support to adult children was still identified as a moderator while receiving instrumental support served the same role only among intensive caregivers.

## Discussion

This study investigated the relationship between grandparenting and depressive symptoms among older Chinese adults, as well as the role of intergenerational support, using a cross-sectional national representative sample from CLASS (2014, 2018).

The first remarkable finding of our study was that grandparenting brought mental health benefits for Chinese older adults. This finding supports Hypothesis 1 but contrasts with Western studies that emphasize the negative health consequences of grandparenting [11, 12, 18, 19, 45]. In the United States, grandparents often assume the responsibility of caring for their grandchildren when adverse family events occur, such as divorce, drug use, or the incarceration of their adult children [46, 47]. Under these circumstances, grandparenting correlated with poorer mental health among older adults. However, in China, traditional cultural norms dictate that grandparents provide childcare in response to their perceived duty toward family development and the continuity of lineage [38]. This understanding often fosters a sense of accomplishment, importance, and self-worth in their role as grandparents, potentially alleviating feelings of insignificance and lack of value that may arise because of aging or retirement.

In addition to existing Chinese studies that emphasize the benefits of low-intensity caregiving [4, 16, 17, 48, 49], we also proposed that high-intensity grandparenting can provide health advantages. This finding may be explained as follows. Firstly, China’s lower fertility rate reduces opportunities for the older adults to become grandparents [37]. High-intensity caregiving allows for increased interaction with descendants, resulting in improved mental health. Secondly, high-intensity care does not exceed the physical and psychological capacity of older adults, as our study only includes older adults who do not have functional impairments. The rewards of caregiving, such as a sense of accomplishment and self-identity, may outweigh the perceived burden, even when the intensity of childcare is high. Findings that link intensive caregiving with poorer mental health or no impact often involve adults over 80 years old [4, 28, 49, 50]. Thus, their outcomes may be more related to inherent health decline rather than the act of caregiving itself.

The second finding of this study indicated that the influence of different types and directions of intergenerational support on grandparents’ mental health was differentiated. Hypothesis 2 was partly supported. Firstly, we observed that stronger emotional support correlates with better mental health among grandparents, consistent with many global studies [33, 39, 51, 52]. Underpinned by Social Convoy Theory, familial interactions provide a crucial support network for elders, thereby preserving their health. Since emotional closeness indicates affective solidarity between generations [53–55], stable and robust emotional bonds suggest more effective resource-support networks for elders, which can help alleviate age-related resource scarcity pressures and enhance mental health.

In terms of financial support, this study found that providing financial assistance to adult children significantly reduced the levels of depression experienced by grandparents. Receiving financial support can improve grandparents’ mental health, but its impact was not significant, which contradicts previous findings [4, 6, 25, 56]. On the one hand, adult children offering economic support to elders adheres to Chinese norms and may be considered a common practice, thus not providing additional psychological benefits [7]. On the other hand, providing economic support for adult children can enhance the autonomy of older people, reduce feelings of dependency, and increase happiness by meeting the needs of their offspring, thereby improving their overall mental well-being [57]. Our findings suggested that understanding intergenerational support in modern China should be based on a model of intergenerational reciprocity, rather than one of equivalent exchange [3, 56, 58]. Since Chinese grandparents receive more financial support from their children than they provide, providing economic support to adult children might have more symbolic significance rather than just a numerical value. The act of economic interaction itself seemed to be of particular importance to grandparents, despite going against Chinese traditional values of upward support transfer [26, 38].

Additionally, regarding instrumental support, we found that providing such support to children can increase grandparents’ levels of depression among grandparents, while receiving it could decrease their depression. However, these results were not statistically significant. For the former, it is possible that grandparents feel more exhausted and stressed due to the additional housework they take on while caring for their grandchildren. This finding differed from the results of Zhang et al. [26]. They found that offering instrumental support to children may enhance the happiness of grandparents. Since they did not differentiate between housework and grandchild care, the protective effect may be attributed to the act of grandparenting. For the latter, a possible explanation might be that even though adult children offering housework support exemplifies filial piety, physically healthy elders might not require it [59].

Furthermore, we have also discovered that providing financial and instrumental support to adult children, as well as receiving economic support from them, significantly impacts the relationship between grandparenting and the depression experienced by older adults. Hypothesis 3 was partially supported. One possible explanation is that providing more support can reduce depression in grandparents by fostering better interaction with their children, promoting family harmony, and enhancing self-worth [12, 41, 60]. However, increased financial support from adult children only enhances the protective effect on mental health in non-intensive childcare situations, with no significant impact on high-intensive childcare situations. This might be because such support, while comforting, cannot fully alleviate the stress of intensive childcare. Our finding indicated that grandparents’ time investment is not in exchange for financial rewards from their adult children, contradicting the ‘time-for-money’ logic [3]. Interestingly, emotional support did not have a significant moderating effect, possibly due to the already strong and stable emotional connections between generations [33].

Indeed, intergenerational care should be seen as a process. During this period, the bi-directional intergenerational interaction will subtly change, which will affect the relationship between grandparenting and the mental health of the elderly. Notably, overlooking the support that grandparents provide to their adult children may result in an oversimplified interpretation of the relationship between grandparenting and the support that adult children provide to the elderly. It is not just a matter of “intergenerational exchange,” where the motivation for grandchild care is obtaining financial support from adult children for a living [59, 61]. This assumption underpins many arguments for intergenerational support as a mediator [61, 62], leading to a lack of examination of intergenerational support’s moderating role. In recent decades, China has undergone significant transformations, including socioeconomic development, population aging, changes in family structures, and adaptations of Chinese traditional norms to modern society. As the socioeconomic status of older individuals improves, their dependence on their children for support has significantly decreased. Older adults are now more capable of offering various forms of support to their adult children, not solely to obtain assistance from their children.

The FE model produced conclusions that were slightly different from the POLS model. Despite the suggestion from the FE model that grandparenting can reduce depression levels in older adults, neither low-intensity nor high-intensity caregiving showed significant effects. This finding aligns with the study conducted by Ku et al. [39]. Differences between the estimations of the POLS and FE models are understandable. Firstly, the FE model only considers within-individual changes, excluding time-invariant characteristics. Secondly, when a substantial number of samples are observed only once in the data, the FE model cannot reliably reflect within-individual variations. Regrettably, 90.78% of our sample fell into these two types of situations. If long-term panel data on grandparenting behaviors of older adults were available in the future, using a FE model would be a more suitable option for capturing the relationship between changes in within-person caregiving behavior and mental health outcomes.

### Strengths and limitations

Compared to existing studies on grandparenting among older Chinese adults, this study has several strengths. First, our study identified that grandparenting, particularly when providing intensive care, can enhance the mental health of older adults. This result confirmed that the advantages of role enhancement outweigh the negative effects of role strain-related detriments in the Chinese grandparenting context. Second, in addition to investigating the support provided by adult children to older people, we also examined the mental health impact of the support provided by older adults to their adult children. Third, we examined the moderating role of different forms of bi-directional intergenerational support on the association between grandparenting and mental health among older adults.

However, our analysis is not without limitations. First, our measurements may not accurately reflect objective health conditions, grandparenting involvement, and intergenerational interactions, as they are based on self-reported data. These factors are sensitive to social regulations and cultural norms. Second, we adopted a rough classification for non-intensive grandparenting, resulting in an insufficient comprehension of non-intensive grandparenting. Third, the CLASS questionnaire did not provide detailed information on grandparenting, such as whether grandparents’ childcare behavior was voluntary or involuntary. Fourthly, this study did not explore potential variations in the correlation between grandparenting and depressive symptoms among subgroups, such as urban-rural and male-female grandparents. Last, we should interpret the analysis results with caution. While we considered several health conditions over time and excluded grandparents with functional limitations, we cannot definitively attribute the results to a causal effect. This is due to the fact that only 32.53% of our study sample in 2014 were observed in 2018 and 90.78% of the total sample were observed only once or lacked variations in grandparenting status. This poses challenges in capturing within-individual changes as our dataset primarily exhibits a cross-sectional rather than a longitudinal nature. Another reason stems from our inability to fully exclude the impact of positive health selection on grandparenting behavior.

## Conclusions

Our study contributed to understanding the grandparenting process, its health effect in the Chinese context, and the role of intergenerational support. Our findings indicated that childcare predicted better mental health in grandparents, especially when they engaged in intensive grandparenting. In addition, we found that emotional closeness and providing financial support to adult children brought mental health benefits to grandparents involved in childcare. Giving financial support and providing instrumental support moderated the association between grandparenting and the mental health of the elderly. However, the moderating role of receiving financial support only exists when non-intensive childcare is provided. Future policies should encourage grandparents in good health conditions to participate in more activities involving the care of their grandchildren. These policies should also promote mutual support among family members, taking into consideration the physical and spiritual needs of the elderly population and the health benefits they can gain from engaging in such activities.

### Electronic supplementary material

Below is the link to the electronic supplementary material.

Supplementary Material 1

Supplementary Material 2

## Data Availability

The data that support the findings of this study are available from the CLASS team (http://class.ruc.edu.cn/.adn) and the Chinese National Survey Data Archive (http://www.cnsda.org/index.php), but restrictions apply to the availability of these data, which were used under license for the current study, and so are not publicly available. Data are, however, available from the authors (Huan Wamg) upon reasonable request and with permission of the CLASS team.

## Abbreviations

*CLASS*: China Longitudinal Aging Social Survey
*POLS*: Pooled ordinary least square
*FE*: Fixed effect model
*COEF*: Coefficient
*CI*: Confidence Interval
*M*: Mean
*SD*: Standard deviation
*ANOVA*: Analysis of variance
