# Supplementary material for: Impacts of grandparenting on older Chinese adults’ mental health: a cross-sectional study
Source: BMC Geriatr. 2023 Oct 13;23:660. doi: 10.1186/s12877-023-04396-x (PMC10571259; doi:10.1186/s12877-023-04396-x)
Supplement: Supplementary file 1 — Supplementary Material 1 [file 12877_2023_4396_MOESM1_ESM.docx]

**Supplemental Table 1**. Fixed effect regression on the association between grandparenting and depressive symptoms, 2014-2018 (Coef. /[CI], N=9486).

| Variables | Model 1 | Model 2 | Model 3 | Model 4 | Model 5 |
| --- | --- | --- | --- | --- | --- |
| **Grandparenting** (ref=no caregiving) |  |  |  |  |  |
| Non-intensive | -0.52 | -0.58 | -0.59 | -0.60 | -0.55 |
|  | [-1.00, -0.03] | [-1.06, -0.11] | [-1.06, -0.11] | [-1.08, -0.11] | [-1.03, -0.07] |
| Intensive | -0.47 | -0.57 | -0.51 | -0.47 | -0.53 |
|  | [-1.29,0.34] | [-1.38,0.24] | [-1.31,0.29] | [-1.31,0.37] | [-1.37,0.30] |
| **Intergenerational support** |  |  |  |  |  |
| Emotional closeness |  | -0.98^***^ |  |  | -1.00^***^ |
|  |  | [-1.63, -0.33] |  |  | [-1.65, -0.35] |
| Giving financial support to adult children (log (+1)) |  |  | -0.07^**^ |  | -0.07^**^ |
|  |  |  | [-0.13, -0.01] |  | [-0.13, -0.00] |
| Receiving financial support from adult children (log (+1)) |  |  | -0.05 |  | -0.04 |
|  |  |  | [-0.13,0.04] |  | [-0.12,0.04] |
| Giving instrumental support to adult children |  |  |  | -0.04 | -0.02 |
|  |  |  |  | [-0.22,0.13] | [-0.20,0.16] |
| Receiving instrumental support from adult children |  |  |  | 0.10 | 0.14 |
|  |  |  |  | [-0.09,0.29] | [-0.05,0.33] |
| Controls | Yes | Yes | Yes | Yes | Yes |
| **Constant** | 4.33^***^ | 7.25^***^ | 5.82^***^ | 5.47^***^ | 7.46^***^ |
|  | [3.07,5.59] | [5.38,9.12] | [4.31,7.32] | [4.07,6.88] | [5.54,9.38] |
| F | 70.33^***^ | 61.72^***^ | 59.28^***^ | 57.26^***^ | 49.97^***^ |

**Note:** Control variables include sex, age group, marital status, living arrangement, residential region, social activity, education, working status, chronic conditions, self-rated health, and wave year. Results were combined using 20 imputed data sets. Coef. = coefficient estimation; Ci= confidence interval; 95% confidence intervals in brackets; ^*^ p < 0.1, ^**^ p < 0.05, ^***^ p < 0.01.
