# Supplementary material for: Impacts of grandparenting on older Chinese adults’ mental health: a cross-sectional study
Source: BMC Geriatr. 2023 Oct 13;23:660. doi: 10.1186/s12877-023-04396-x (PMC10571259; doi:10.1186/s12877-023-04396-x)
Supplement: Supplementary file 2 — Supplementary Material 2 [file 12877_2023_4396_MOESM2_ESM.docx]

**Supplemental Table 2.** Fixed effect regression on interaction effect of intergenerational support on the association between grandparenting and depressive symptoms, 2014-2018 (Coef. /[CI], N=9486)

| Variables | Model 6 | Model 7 | Model 8 | Model 9 |
| --- | --- | --- | --- | --- |
| **Grandparenting** (ref=no caregiving) |  |  |  |  |
| Non-intensive | -0.36 | -1.43^**^ | -0.91^**^ | -1.00 |
|  | [-3.18,2.45] | [-2.75, 0.12] | [-1.70, 0.11] | [-3.96, 1.96] |
| Intensive | -1.65 | 0.13 | 0.74 | -1.93 |
|  | [-5.82,2.52] | [-1.79,2.04] | [-0.51,1.98] | [-5.95,2.09] |
| **Interactions** |  |  |  |  |
| Non-intensive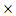Emotional closeness | 0.10 |  |  | 0.36 |
|  | [-1.37,1.56] |  |  | [-1.11,1.83] |
| Intensive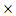Emotional closeness | 0.59 |  |  | 1.53 |
|  | [-1.57,2.74] |  |  | [-0.57,3.63] |
| Non-intensive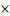Giving financial support to adult children (log (+1)) |  | -0.17^***^ |  | -0.16^**^ |
|  |  | [-0.29, -0.05] |  | [-0.28, -0.03] |
| Intensive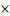Giving financial support to adult children (log (+1)) |  | -0.30^***^ |  | -0.26^***^ |
|  |  | [-0.49, -0.11] |  | [-0.45, -0.07] |
| Non-intensive 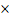Receiving financial support from adult children (log (+1)) |  | -0.07 |  | -0.07 |
|  |  | [-0.25,0.12] |  | [-0.26,0.11] |
| Intensive 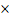Receiving financial support from adult children (log (+1)) |  | 0.06 |  | 0.07 |
|  |  | [-0.19,0.30] |  | [-0.18,0.31] |
| Non-intensive 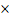Giving instrumental support to adult children |  |  | -0.30 | -0.24 |
|  |  |  | [-0.69,0.09] | [-0.64,0.16] |
| Intensive 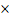Giving instrumental support to adult children |  |  | 0.01 | 0.21 |
|  |  |  | [-0.50,0.52] | [-0.31,0.74] |
| Non-intensive 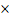Receiving instrumental support to adult children |  |  | -0.06 | -0.02 |
|  |  |  | [-0.45,0.32] | [-0.42,0.37] |
| Intensive 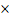Receiving instrumental support to adult children |  |  | -0.91^***^ | -0.98^***^ |
|  |  |  | [-1.43, -0.40] | [-1.50, -0.47] |
| Controls | Yes | Yes | Yes | Yes |
| Constant | 7.64^***^ | 7.10^***^ | 7.13^***^ | 7.46^***^ |
|  | [4.97,10.31] | [5.07,9.14] | [5.17,9.10] | [4.77,10.15] |
| F | 44.69^***^ | 41.47^***^ | 42.44^***^ | 33.66^***^ |

**Note:** Control variables include sex, age group, marital status, living arrangement, residential region, social activity, education, working status, chronic conditions, self-rated health, and wave year. Results were combined using 20 imputed data sets. Coef. = coefficient estimation; CI = confidence interval; 95% confidence intervals in brackets; ^*^ p < 0.1, ^**^ p < 0.05, ^***^ p < 0.01.
